# Comparison of Compression-Based Measures with Application to the Evolution of Primate Genomes

**DOI:** 10.3390/e20060393

**Published:** 2018-05-23

**Authors:** Diogo Pratas, Raquel M. Silva, Armando J. Pinho

**Affiliations:** 1 Institute of Electronics and Informatics Engineering of Aveiro, University of Aveiro, 3810-193 Aveiro, Portugal; 2 Department of Medical Sciences and Institute for Biomedicine - iBiMED, University of Aveiro, 3810-193 Aveiro, Portugal; 3 Department of Electronics, Telecommunications and Informatics, University of Aveiro, 3810-193 Aveiro, Portugal

**Keywords:** data compression, NCD, NRC, DNA sequences, primate evolution

## Abstract

An efficient DNA compressor furnishes an approximation to measure and compare information quantities present in, between and across DNA sequences, regardless of the characteristics of the sources. In this paper, we compare directly two information measures, the Normalized Compression Distance (NCD) and the Normalized Relative Compression (NRC). These measures answer different questions; the NCD measures how similar both strings are (in terms of information content) and the NRC (which, in general, is nonsymmetric) indicates the fraction of one of them that cannot be constructed using information from the other one. This leads to the problem of finding out which measure (or question) is more suitable for the answer we need. For computing both, we use a state of the art DNA sequence compressor that we benchmark with some top compressors in different compression modes. Then, we apply the compressor on DNA sequences with different scales and natures, first using synthetic sequences and then on real DNA sequences. The last include mitochondrial DNA (mtDNA), messenger RNA (mRNA) and genomic DNA (gDNA) of seven primates. We provide several insights into evolutionary acceleration rates at different scales, namely, the observation and confirmation across the whole genomes of a higher variation rate of the mtDNA relative to the gDNA. We also show the importance of relative compression for localizing similar information regions using mtDNA.

## 1. Introduction

In 1965 [1], Kolmogorov described three ways to measure the information contained in strings: combinatorial [2,3], probabilistic [4] and algorithmic. The algorithmic approach, also known as Kolmogorov complexity or algorithmic entropy, enables measurement and comparison of the information (or complexity) contained in different natural processes that can be expressed using sequences of symbols (strings) from a finite alphabet [1,5,6,7,8,9,10,11,12,13].

The Kolmogorov complexity differs from the Shannon entropy [4], because it considers that the source, rather than generating symbols from a probabilistic function, creates structures that follow algorithmic schemes [14,15]. Therefore, to reverse the problem, there is the need to identify the program(s) and parameter(s) that generate the outcome(s) [1,11]. Successful implementations, using small Turing machines [16], have been proposed and implemented (for example [17,18]). Other implementations, using lossless data compressors, have also been proposed [19,20].

The Kolmogorov complexity is non-computable [21], mostly because of the halting problem [22]. Therefore, we have to rely on approximations such as string compressors, C(x). For a definition of safe approximation, see [23]. The normalized version, known as the Normalized Compression (NC), is defined by

(1) $$\mathrm{NC}\left(x\right)=\frac{C(x)}{\left|x\right|\log_{2}\left|A\right|},$$

where *x* is a string, |A| the number of possible different elements in *x* (size of the alphabet) and |x| the length of *x*. The NC enables to compare the information contained in the strings independently from their sizes [19].

The usage of compressors has also been applied to approximate the amount of information between two strings, *x* and *y*, namely through the Normalized Compression Distance (NCD) [11,24,25,26,27,28]. In order to compute this distance, we may use the conditional compression [29,30], C(x|y), as

(2) $$\mathrm{NCD}\left(x,y\right)=\frac{\max\{C(x|y),C(y|x)\}}{\max\{C(x),C(y)\}},$$

or the conjoint compression [25], C(x,y), as

(3) $$\mathrm{NCD}\left(x,y\right)=\frac{C(x,y)-\min\{C(x),C(y)\}}{\max\{C(x),C(y)\}},$$

given that the conditional and conjoint compressions are related through the chain rule [11].

The NCD enables to measure the information between two strings, being robust to some degree of noise [31], as long as the compressor respects the normality properties [11,32].

There are many examples of the NCD applicability, namely in plagiarism analysis [33], stemmatology [34], DNA analysis [27,28,35,36,37], gene expression analysis [38,39], complex system behaviour studies and time series analysis [40], image distinguishability [41], image similarity [42,43,44], image distortion analysis [45], visual analysis of document collections [46], classification of file fragments [47], retinal progenitor cell fate prediction and handwritten digits classification [48], clustering music [49], evolving computer-generated music [50], cover songs identification [51], entity identification [52], assessing the impact of students in peer review [53], analysis of internet malware [54,55], analysis of software systems stability [56], measuring the similarity between black-and white-box regression test prioritization techniques [57], optimization of compilers [58], analysis of public opinion [59], deception detection [60], and fawns detection [61].

On the other hand, to measure the information of a string relative to another [62,63], we have to rely on relative semi-distances. These are measures that do not need to respect two distance properties, namely symmetry and the triangle inequality [64]. Several approaches to quantify the relative information have been proposed (e.g., [62,65,66,67,68,69]), such as for handling images [66], texts [68,69], ECG data [70], and genomic sequences [71,72,73].

The relative information can be approximated using relative compressors [62,68,69,72,74,75,76,77], regardless if they are based on dictionaries [62,68,74] or Markovian models [69,72]. These compressors aim to model and organize the data of *y* (without knowing *x*). Then, they freeze the model of *y* [78] and, finally, they measure the number of bits needed to describe *x*, using exclusively the information from *y*. We call this operation the compression of *x* relatively to *y* and denote it by C(x∥y).

The information of *x* relative to *y*, C(x∥y), is a sum of the information content provided, symbol by symbol, after processing the complete *x*, according to

(4) $$C\left(x∥y\right)=\sum_{i=1}^{\left|x\right|}C\left(x_{i}∥y\right),$$

where |x| is the size of string *x*. The information profile of $C(x_{i}∥y)$ is very important to localize regions that are similar in *x* relatively to *y*, namely through the lower information content regions below a certain threshold [64,71]. For overall quantities, we need to compute the Normalized Relative Compression (NRC) as

(5) $$\mathrm{NRC}\left(x∥y\right)=\frac{C(x∥y)}{\left|x\right|\log_{2}\left|A\right|}.$$

The NRC enables to compare and relate symbolic sequences, namely DNA sequences, permitting to test multiple hypotheses and infer patterns in most of the evolution theories [64].

The main theories in evolution shown that species evolve and have a common ancestral [79,80,81,82]. This means that the DNA between close species is very similar [81,82]. Therefore, the lower the variation among the species, the lower the information needed from one species DNA sequence to relatively describe the other.

However, we should note that, in practice, genomic sequences are not just a succession of letters with four possible outcomes (A,C,G,T), which indicate the order and nature of nucleotides within a DNA chemical chain. They are also the outcome of machines and algorithms due to the sequencing and assembling phases [83]. In reality, they are the outcome of a probabilistic capture of small pieces from a huge puzzle with lots of repeated, changed and missing pieces [84,85].

Additionally, genomic sequences have other alteration sources, for example environmental factors [86,87], pathogen species [88,89], rearrangements [90,91], and unknown sources [92]. Therefore, dealing with DNA sequences from different species is the equivalent of dealing with heterogeneous, dynamic, incomplete and imperfect information [93].

In eukaryotic cells, DNA sequences can have different sources, namely from the nucleus and organelles [94]. In animals, the double membrane-bound organelles are mitochondria, while in plants are mitochondrion and chloroplasts (plastids). The nuclear sequence (gDNA) is substantially longer than the mitochondrial sequence (mtDNA). A recent hypothesis to the conservation of the mtDNA size has been pointed to the protection against virulence agents [95].

The quantity of mutations that have been accumulated between two genomes provides a record of the time elapsed since their common ancestor [96]. The NCD and NRC are measures that are able to quantify variation, particularly because they measure information between and across DNA sequences. Moreover, when using relative measures, we can extend it to profile the information content enhancing relations and correlations between the data [71].

In this paper, we directly compare the NCD and NRC in synthetic and real data. For the purpose, we use a state-of-the-art compression algorithm and apply it on DNA sequences with different scales and natures. First, we compare the measures in synthetic data, with different characteristics. Then, we apply them on mtDNA, mRNA, and gDNA of seven primates. Finally, we show the importance of relative compression for localizing similar information regions using the mtDNA.

## 2. Methods

All the results provided in this paper can be replicated using the scripts provided in the repository https://github.com/pratas/APE. These will have as a dependency the GeCo compressor [72]. GeCo is freely available, under GPLv3 license, at https://github.com/pratas/geco. GeCo is a DNA sequence compressor, with state of the art compression capabilities, and uses reasonable computation resources. As an alignment-free tool [97,98], GeCo is able to determine absolute measures, namely for many (semi) distance computations, and local measures, such as the information content associated with each element. GeCo allows both reference-free and relative compression. Therefore, all the compression results provided in this paper were obtained using GeCo, although with custom models and parameters.

### 2.1. Compressor and Parameters

We use GeCo to compute the NCD (Equation (3)) and NRC (Equation (5)). For these two measures we only use two types of compression, C(x) and C(x∥y), mainly because the conjoint compression in Equation (3), C(x,y), can be approximated using the compression of *x* concatenated with *y*, namely C(x,y)≈C(xy)=C(yx), as long as the *normality* properties are verified [32,99]. Notice that C(xy) stands for the compression of *x* and *y* concatenated together, while C(x,y) represents the compression of *x* and *y*, as well as the necessary description to split them apart (split xy into *x* and *y*). Since the number of bits needed to represent a program to split xy is asymptotically insignificant, we can consider that the approximation is reasonable.

The GeCo compressor enables parametrization and a choice of the models for the two different modes needed, C(x) and C(x∥y). Despite the compression mode, the compressor uses a cooperation between multiple context models and substitutional tolerant context models [72,100].

**Figure 1:**
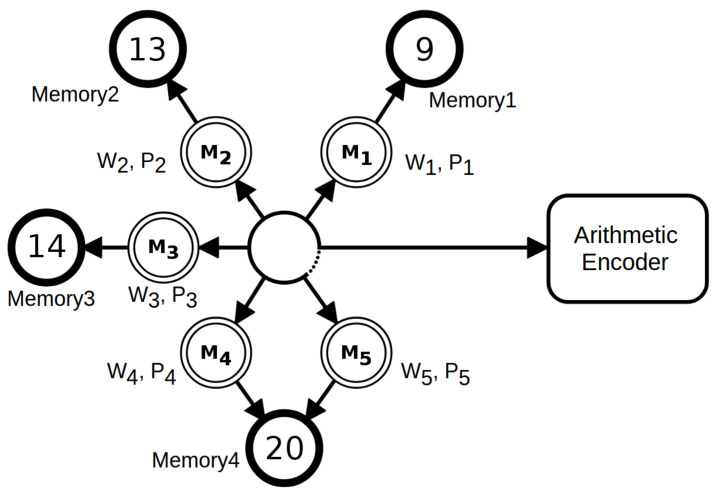
A mixture of five context models. Each model has a weight (*W*) and associated probabilities (*P*) that are calculated according to the respective memory model. The tolerant context model (5) uses the same memory of model 4, since they have the same context (depth 20). After, the probabilities are averaged according to the respective weight and redirected to the arithmetic encoder.

The cooperation is supervised by a soft blending mechanism [101,102] that gives importance to the models that had a better performance given an exponential decayment record [102]. Figure 1 depicts an example of the cooperation between five context and tolerant context models, namely $M_{1},\;M_{2},\;M_{3},\;M_{4},\;M_{5}$. Each of these models has a probability (*P*) and a weight (*W*) associated.

The difference between the C(x) and the C(x∥y) is given by the initialization and access to the memory models. In C(x), the memory model starts with uniform counters, each set to zero. Through all the computation of C(x) the memory model is updated. On the other hand, the C(x∥y) is initialized with the memory of *y*, then the memory model is set static and used through all the computation of *x*.

The probability of each symbol, $x_{i}$, is given by

(6) $$P\left(x_{i}\right)=\sum_{m\in M}P_{m}\left(x_{i}|x_{i-k}^{i-1}\right)\;w_{m,i},$$

where $P_{m}\left(x_{i}|x_{i-k}^{i-1}\right)$ is the probability assigned to the next symbol by a context or substitutional tolerant context model, *k* the order of the corresponding model *m*, and where $w_{m,i}$ denote the corresponding weighting factor, with

(7) $$w_{m,i}\propto {\left(w_{m,i-1}\right)}^{\gamma_{m}}P_{m}\left(x_{i}|x_{i-k}^{i-1}\right),$$

where $\gamma_{m}\in \left[0,1\right)$ acts as a forgetting factor for each context model. Frequently, we use the same value for each $\gamma_{m}$, since its optimization only provides minimal gains. The sum of the weights, for all the respective models, must be equal to one.

The depth of the model, *k*, identifies the number of contiguous symbols seen in the past for predicting the next symbol and, hence, $x_{i-k}^{i-1}$ [103]. The alpha is an estimator parameter that allows balancing between the uniform and the frequency distribution (usually deepest models have lower alphas [104]). The inverted repeats define if a short program, that allows searching for subsequences with similarity in complemented reverse sequences, is running (normally used in deepest models). The tolerance is a short program that enables to set the number of allowed substitutions in a certain context depth [72,100]. The cache-hash enables to keep in memory only the latest entries up to a certain number of hash collisions [99].

We use GeCo with a set of models and parameters according to the length and nature of the data, that have been set according to our experience. Notice that better models may be achieved with optimization, although we believe that the precision is good enough. The parameters are the following:

- C(x∥y)→ mixture of seven models with a decayment (γ) of 0.95 and a cache-hash of 30:
  1 tolerant context model: depth: 17, alpha: 0.02, tolerance: 5;
  2 context model: depth: 17, alpha: 0.002, inverted repeats: no;
  3 tolerant context model: depth: 14, alpha: 0.1, tolerance: 3;
  4 context model: depth: 14, alpha: 0.005, inverted repeats: no;
  5 context model: depth: 11, alpha: 0.01, inverted repeats: no;
  6 context model: depth: 8, alpha: 0.1, inverted repeats: no;
  7 context model: depth: 5, alpha: 1, inverted repeats: no;
- C(x) and C(xy)→ mixture of eight models with a decayment (γ) of 0.95 and a cache-hash of 30:
  1 tolerant context model: depth: 17, alpha: 0.1, tolerance: 5;
  2 context model: depth: 17, alpha: 0.005, inverted repeats: no;
  3 tolerant context model: depth: 14, alpha: 1, tolerance: 3;
  4 context model: depth: 14, alpha: 0.01, inverted repeats: no;
  5 context model: depth: 11, alpha: 0.1, inverted repeats: no;
  6 context model: depth: 8, alpha: 1, inverted repeats: no;
  7 context model: depth: 5, alpha: 1, inverted repeats: no;
  8 context model: depth: 3, alpha: 1, inverted repeats: no.

### 2.2. NCD versus NRC in Synthetic Data

**Figure 2:**
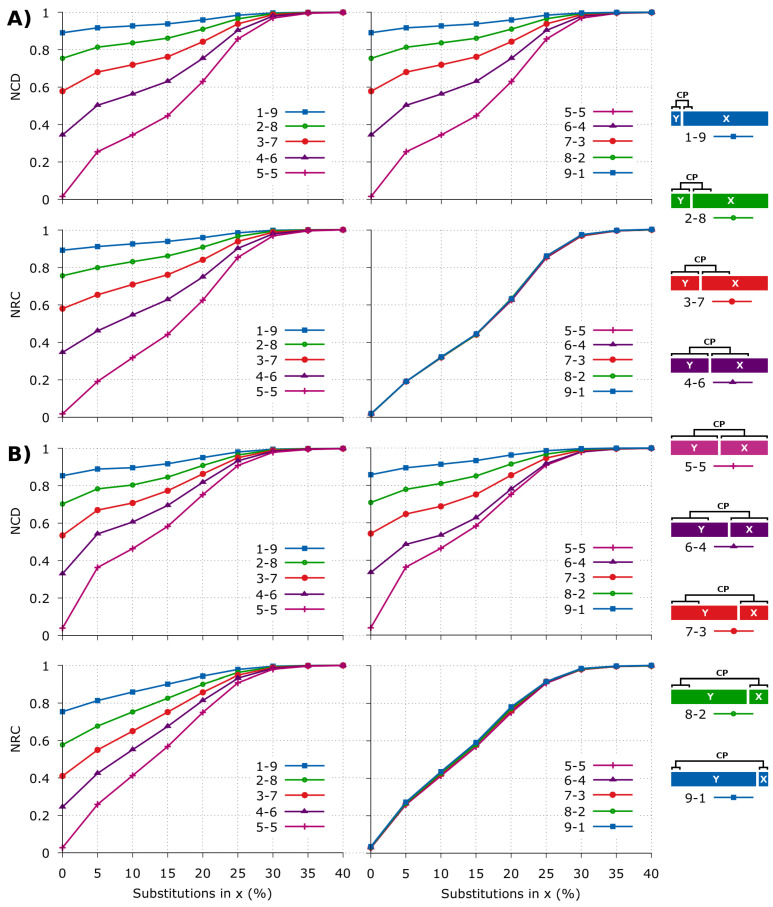
Comparison of the NCD (Equation (3)) and the NRC (Equation (5)) for several synthetic sequences with different substitutions applied on *x*. The sequences architecture is at right, where “CP” means copy. The substitutions in *x* are only applied after coping a region of *y* into *x*. Each pair, *x* and *y*, has a length of 1 MB. (**A**) the distribution of the sequences is uniform. For replication use the script runComparison.sh; (**B**) distribution is not uniform, and the sequences contain multiple repeats [105]. The numbers *a*-*b* stand for string sizes proportions, for example 1-9 means that *y* has size 0.1 MB and *x* 0.9 MB. For replication use runComparisonWithRedundancy.sh.

Using the mentioned compressor, parameters and modes, we have compared the NCD and the NRC in synthetic data with custom redundancy, rearrangement formats and mutation rates. The sequences have been built using copies with different sizes and mutation rates, according to the legend (right panel) of Figure 2.

As it can be seen (Figure 2), there is an approximate symmetry in the NCD (Figure 2A). On the other hand, the NRC spends approximately the same amount of information to describe the *x* that have been originally extracted from *y*, regardless the rate of substitutions applied (when |x|≤|y|).

When the distribution of the synthetic sequences is not uniform, being more similar to real DNA sequences (simulated with XS [105]), we are able to notice a decrease in the amount of information needed to describe *x* (Figure 2B), as well as higher values in the NCD in comparison with the NRC. This characteristic enables to predict higher NCD values on real datasets. On the other hand, comparing Figure 2A with Figure 2B, we see an increase in the non-linear behaviour of the NCD, while the NRC seems to approximate to a linear behaviour.

The most significant computations in the NCD are the C(x) and C(yx), while for the NRC is the C(x∥y). Namely, because the normalization of the NRC is given by the size of *x* times a constant (2). Therefore, we now look into to the profiles of each computation in order to compare them. We emphasize that the NCD can be computed using the conjoint (Equation (3)) or conditional (Equation (2)) compression, and, therefore, C(x|y)=C(yx)-C(y), an approximation that can be made through the chain rule [11] (as previously mentioned).

**Figure 3:**
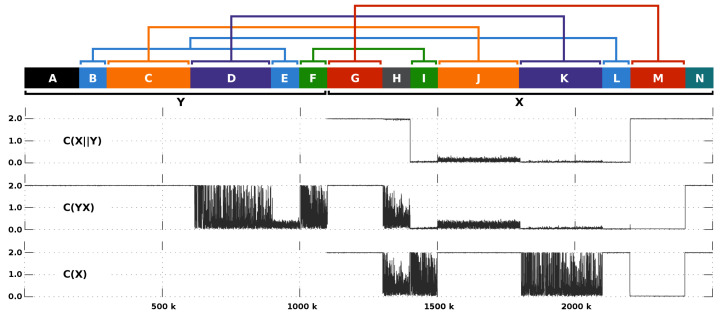
Comparison of the C(x∥y) (top profile), the C(yx) (middle profile) and the C(x) (bottom profile) given several types of rearrangements between *x* and *y*. The upper map depicts the multiple block regions that compose *x* and *y*. The region A and N identify unmatched sequences with high entropy, while H an unmatched sequence with low entropy. Region E and L are a copy of B (high entropy), both with 1% of substitutional mutations. Region J is a copy of C (high entropy) with 1% of substitutional mutations. Region K is a copy of D, both with low entropy. Region I is a copy of F, both with low entropy. Region M is a copy of G, both with high entropy. The sequences have been generated using XS [105] and GOOSE (https://github.com/pratas/goose). For replication use runLocalMethod.sh.

Using this connection, we have simulated two sequences, *x* and *y*, with characteristics according to Figure 3. Then, we have computed and compared the profiles of C(x), C(yx) and C(x∥y).

In Figure 3 we notice that, contrary to C(x) and C(yx), the C(x∥y) is not able to measure similar sub-regions in *x*, namely because the region M, which is a copy of G, is measured as a high complexity region. This is what it means to use the exclusive information from *y*. In fact, ignoring constants, we are able to asymptotically generalize this as

(8) $$C\left(x^{n}∥y\right)=nC\left(x∥y\right),$$

where $x^{n}$ is the concatenation of *n* copies of *x*.

Additionally, we are able to notice that C(x∥y) better describes the region J than C(yx). This might be due to the parameter alpha that is more appropriate for the relative compression mode and because of the model memory of C(x∥y) is lower than C(yx). The last is given the fact that C(yx), besides modelling *y*, adds the events seen in *x*, while the C(x∥y) only maps *y*.

## 3. Results

In this section, we use the NC, NCD, and NRC to measure the information in, between and across mtDNA, mRNA, and gDNA of seven primates. For the purpose, we provide the description of the primate dataset and the parameters used in the compressor as well as a benchmark of the compressor in different compression modes, then we make some previsions applying alterations on the datasets and, finally, provide the empirical results.

All the results presented in this paper can be reproduced, under a Linux OS, using the scripts provided at the repository https://github.com/pratas/ape, specifically runNC.sh, runNCD.sh, runNRC.sh, runReferenceFreeComparison.sh, runReferenceFreeConjoint.sh, runRelativeCompressors Comparison.sh, runExpectationNRC.sh, and runRearrange.sh.

### 3.1. Dataset

We have used the mtDNA, mRNA, and gDNA of seven primates according to the Table 1.

### 3.2. Parameters

The models and parameters used in the compressor are fundamental to ensure compression efficiency. The optimization of the parameters provides better compression results and, therefore, the minimum of a compression result is an indicator of the best-known set of parameters for a specific dataset. However, the optimization requires computational time that, given the length of the larger sequences, becomes prohibitive. For these results, we use a set of models and parameters built upon our experience. The parameters used for the compression of the primates DNA sequences, for the respective nature of the data, are the following:

- **mtDNA**→ mixture of five models with a decayment (γ) of 0.95:
  1 tolerant context model: depth: 13, alpha: 0.1, tolerance: 5;
  2 context model: depth: 13, alpha: 0.005, inverted repeats: yes;
  3 context model: depth: 10, alpha: 0.01, inverted repeats: yes;
  4 context model: depth: 6, alpha: 1, inverted repeats: no;
  5 context model: depth: 3, alpha: 1, inverted repeats: no;
- **mRNA**→ mixture of seven models with a decayment (γ) of 0.88 and a cache-hash of 200:
  1 tolerant context model: depth: 20, alpha: 0.1, tolerance: 5;
  2 context model: depth: 20, alpha: 0.005, inverted repeats: yes;
  3 context model: depth: 14, alpha: 0.02, inverted repeats: yes;
  4 context model: depth: 13, alpha: 0.05, inverted repeats: no;
  5 context model: depth: 11, alpha: 0.1, inverted repeats: no;
  6 context model: depth: 9, alpha: 1, inverted repeats: no;
  7 context model: depth: 4, alpha: 1, inverted repeats: no;
- **gDNA**→ mixture of six models with a decayment (γ) of 0.88 and a cache-hash of 250:
  1 tolerant context model: depth: 20, alpha: 0.1, tolerance: 5;
  2 context model: depth: 20, alpha: 0.005, inverted repeats: yes;
  3 context model: depth: 14, alpha: 0.02, inverted repeats: yes;
  4 context model: depth: 13, alpha: 0.05, inverted repeats: no;
  5 context model: depth: 11, alpha: 0.1, inverted repeats: no;
  6 context model: depth: 9, alpha: 1, inverted repeats: no.

For the relative compression the parameters and models have been the same, with the exception of the alpha for context 14 (set to 0.01) and that the models were in the beginning loaded with the counts regarding *y* and, then, set static through all the computation of *x* (described in Section 2.1). The maximum RAM used was 34.3 GB (for the gDNA dataset, which has an approximate sum of 18 GB). Essentially, by a small precision payoff the RAM might be decreased, despite in this experience we wanted to cope with the full *normality* properties, namely the idempotency [32].

### 3.3. Comparison of Compressors

The NCD and NRC measures are extremely dependent on the capability for the compressor to reduce losslessly the storage associated with the respective sequences, independently if the running mode is reference-free or relative. The better the compression is, the more reliable are the results. This happens because the compressor acts as a description program of a string given what it knowns in each specific moment with the extra information (from *y*) when it is available. If the compressor is not efficient while describing the strings, then the computation of the measure will not be accurate, sometimes even showing misleading results. On the other hand, if the compressor is prepared to handle most of the characteristics of the data, such as in this case, genomic rearrangements (translocations, inversions, duplications, fissions, fusions), stochastic variation (especially, high level of substitutions), and high heterogeneity (high alteration between high and low complexity regions), then it is an efficient describer and an obvious candidate to be used in the NCD and NRC measures. Therefore, it is not just to cope with the normality characteristics, but also to be as efficient as possible for the data type.

The interest for DNA sequence compression was started with the Biocompress algorithm in 1993 [106]. The subsequent two and half decades have seen the publication of a considerable number of algorithms for reference-free compression of DNA sequences (e.g., [72,102,107,108,109,110,111,112,113,114,115,116,117,118,119,120,121,122,123,124,125,126,127,128,129]) and for reference-based compression (e.g., [72,75,76,77,118,126,130,131,132,133,134,135,136,137]). With the development of the next-generation sequencing and the increasing availability of genomic data [138], several compression algorithms have been developed to cope with the specific needs of special file formats, namely the inclusion of multiple information channels, for example, quality-scores, as well as adding increasing levels of redundancy [139,140,141,142,143,144,145,146,147,148]. For a review of biological sequence compression algorithms, see [149].

In order to guarantee that GeCo is an efficient and appropriate compressor for the task, we compare GeCo with some of the best known high-ratio reference-free and relative compressors. However, notice that since, in this paper, we also use large-scale sequences (several GB), and to cope with the normality, we excluded the usage of XM [118]. The XM method uses computational resources that are not affordable to use in a large-scale scenario. Nevertheless, it is worth to mention that we have found competitive results and, sometimes, marginal higher compression capabilities in XM relative to GeCo.

**Figure 4:**
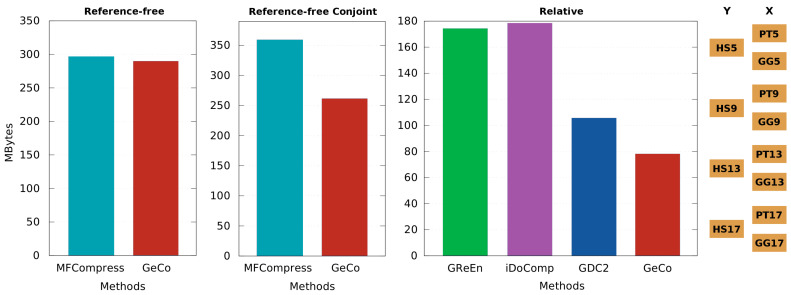
Number of MegaBytes needed for each compression tool to represent a lossless compact form of each dataset. Benchmark for three types of compression is provided: reference-free (C(x)), reference-free conjoint (C(yx)) and relative (C(x∥y)). The reference-free includes the compression of chromosome sequences corresponding to HS5, PT5, GG5, HS9, PT9, GG9, HS13, PT13, GG13, HS17, PT17, GG17. The reference-free conjoint, the HS5_PT5, HS5_GG5, HS9_PT9, HS9_GG9, HS13_PT13, HS13_GG13, HS17_PT17, HS17_GG17. The relative, the PT5-HS5, GG5-HS5, PT9-HS9, GG9-HS9, PT13-HS13, GG13-HS13, PT17-HS17, GG17-HS17. The prefix initials stand for species (HS→human, PT→chimpanzee, GG→gorilla), the “_” stand for concatenation, and “-” for “relative to”. For replication use scripts runReferenceFreeComparison.sh, runReferenceFreeConjoint.sh and runRelativeCompressorsComparison.sh.

Figure 4 depicts a benchmark for several chromosome sequences of a human, chimpanzee and gorilla. As it can be seen, GeCo, with the models and parameters described in the previous subsection, with the exception of a cache-hash set to 50 (more appropriate to the size of the samples), achieved always the best compression results, namely 3.16% and 27.2% compression improvement over MFCompress in reference-free compression and reference-free conjoint compression (respectively), and 26% over GDC2 (the second best). The compression gain is calculated with 100(a-b)/a, where *a* stands for the number of bytes compressed with the second best compressor and *b* for the number of bytes compressed with GeCo.

Although we have used four chromosome sequences of each human, chimpanzee and gorilla references, we believe that the compression gain will increase for chromosome sequences of more distant species, namely baboon and marmoset. The reason is that GeCo uses multiple Markov and Tolerant Markov models in cooperation and, hence, it is more suitable to dissimilar species relative to the other compressors. Of course, the penalty is computational resources, namely memory and time. Regarding memory, GeCo needed 9 GB of RAM and used five times the time of the average compressors. Nevertheless, since the objective is to measure dissimilarity as best as we can, we believe that, currently, these resources are affordable to be used. Moreover, GeCo enables to set the models according to the available memory of the machine, independently of the size of the dataset to be compressed and respective reference. This includes the capability to be used, with good results, in even larger scale scenarios.

### 3.4. Expectation

**Figure 5:**
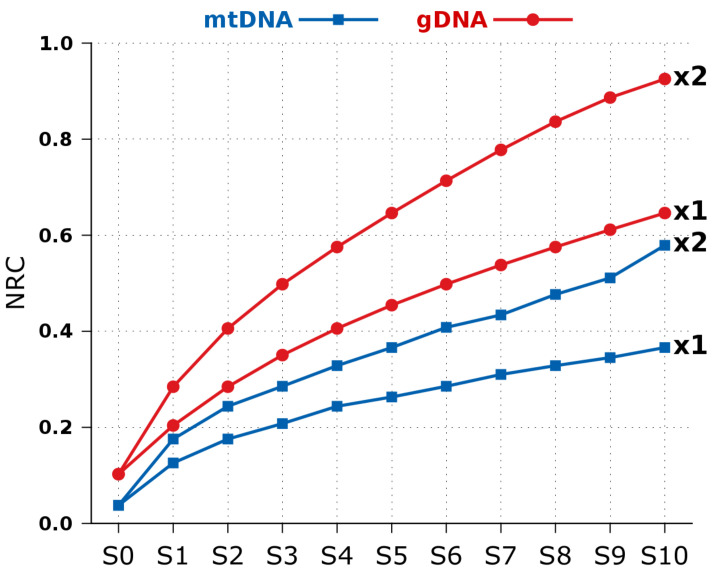
Normalized Relative Compression (Equation (5)) for several substitutional mutations applied to the human mtDNA and gDNA. The “x1” identifies the slope of the mutation rate of 1, while “x2” a 2%. The mutation rate at a given point is identified by multiplying the suffix number by the slope. For replication use runExpectationNRC.sh.

In order to understand how the NRC behaves according to substitutional mutations using very different lengths, we have computed an experiment using the real mtDNA and gDNA of the human as a starting point and, then, we have applied different substitutional mutations rates to several copies of the original. Finally, for the respective scale (mtDNA or gDNA), we have computed the NRC using, as *x*, every copy and, as *y*, the human reference. Figure 5 depicts the result.

As it can be seen, the scale provides a different slope between the constant increasing of substitutional mutations. In this example, the slope two (x2) in the mtDNA seems more similar to the slope one (x1) in the gDNA. Notice that, unlike in the gDNA, the “x2” of the mutated mtDNA has more noise because the sequence has near only 16,000 symbols, which makes it more sensible and prone to collisions in pseudo-random attributions in the process of synthetically creating substitutional mutations.

### 3.5. Primate Analysis

**Figure 6:**
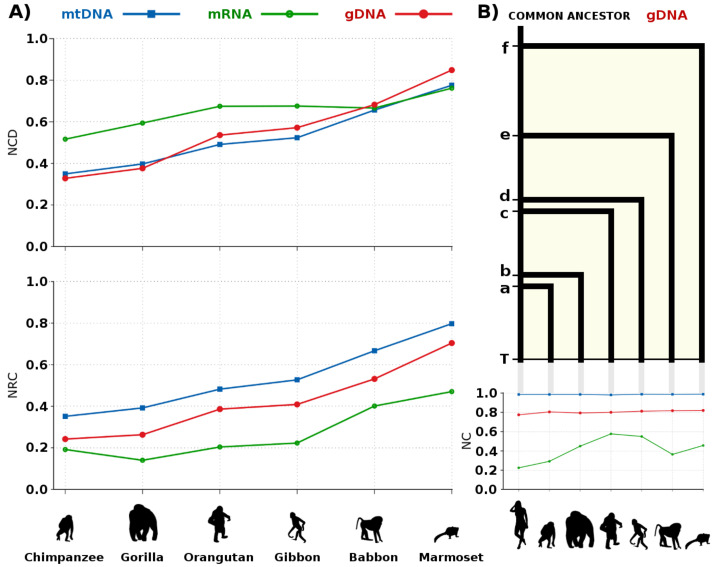
(**A**) Normalized Compression Distance (NCD at the upper plot, using Equation (3)) and Normalized Relative Compression (NRC at the lower plot, using Equation (5)) of mtDNA, mRNA, and gDNA sequences for several anthropoids in relation to the human genome. The gDNA represents the whole genome, including the unplaced and unlocalized sequences, with exception of the Y chromosome (female species); (**B**) Evolutionary tree of the gDNA is up to scale, based on the NRC. Letters a, b, c, d, e, and f represent the divergence time between the respective species, while T stands for the actual time. The NRC of the human relatively to the human has been subtracted from each result (≈0.1). All the genomes have been sequenced in T. The bottom right plot represents the Normalized Compression (NC, using Equation (1)) for each species. For replication use the scripts: runNCD.sh, runNRC.sh and runNC.sh.

In Figure 6, we provide the NCD and NRC for six primates, setting as *y* the human. The main idea was to use the highest quality sequence (human) as a reference in the NRC and because in Figure 2 we have seen that the NRC provides better comparative results when |x|<|y|. As expected the mtDNA and gDNA information values, both in the NCD and NRC, increase as the divergence of the species increase, since the genomes of the primates were ordered given the divergence time depicted in the common literature [150].

**Figure 7:**
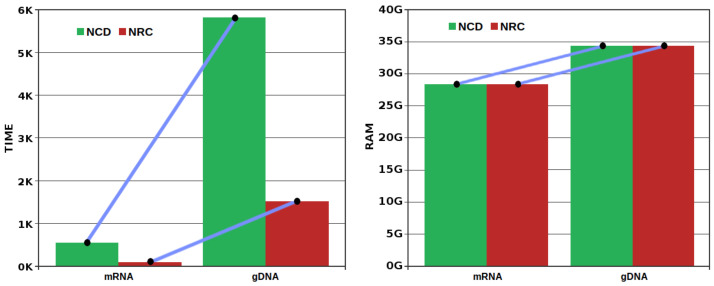
Time (**left**), in minutes, and RAM (**right**), in Gigabytes, needed to compute the NCD and NRC, for the mRNA and the gDNA, in all the measures of Figure 6A. The computation for the mtDNA spent only a few seconds and used less than 0.5 GB of RAM. Given the present orders of magnitude, is asymptotically irrelevant, and, hence, we have excluded from this image. The RAM needed to compute both measures was equivalent for each data type. All the computations were performed in a single core at 2.13 GHz (without parallelization). Unlike the NCD, the NRC can be easily parallelized while maintaining approximately the same RAM using an efficient speed-up.

In Figure 7, we show the cumulative time and maximum RAM needed to compute the mRNA and the gDNA for the respective measures (computation of Figure 6A). The time needed to compute the NRC is much lower than the NCD, especially in the larger dataset (gDNA) where it spent, roughly, one-fifth of the NCD. Notice that both computations used approximately the same RAM (for example, 34.3 GB in the largest dataset). We recall, that lower RAM may be used although having at some point impact in the precision of the measure. Although in this paper we have not studied this subject, this subject would be a thematic of a very interesting work.

Notice that both NCD and NRC are measuring the complete or whole information of the samples, that in the case of the gDNA is not a sampling of the genome, namely only the genes sequences but rather the entire DNA, such as the coding and non-coding regions. Besides, both are able to efficiently deal in an unsupervised mode with chromosomal rearrangements (translocations, inversions, duplications, fissions, fusions), high stochastic variation (especially high level of substitutions) and high heterogeneity of the data (high alteration between high and low complexity regions). This is something, as far as we know, that alignment-based methods are not able to cope using the reported times [97,98,151]. Moreover, the RAM necessary to work with two genomes is approximately the same as with 20. Besides, the computational time increases linearly with the number of input genomes.

Regarding the meaning results, we notice that the NRC for the mRNA of the gorilla is lower than for the chimpanzee. This could indicate that when compared to the human genome, parts that are more similar to the gorilla than to the chimpanzee. However, we have to take into account that the gorilla mRNA sequence has approximately half the size of the chimpanzee mRNA sequence and lower redundancy (NCBI source), as depicted by the NC.

On the other hand, the NCD shows that the information between the human-chimpanzee is lower than human-gorilla, which is according to the well-known theories in primates evolution [152,153,154]. There is also the hypothesis of hybridization [154], such as in the colobine monkeys, although unlikely, given that the NRC of the mtDNA and gDNA shows the opposite behaviour. Additionally, we recall that these sequences, besides having duplications [155,156], are outcomes of processing algorithms [157,158], that need to perform alignments with the gDNA and, therefore, they are dependent on pattern matching algorithms.

The mtDNA and gDNA are sequences which, given their nature, offer a more reliable and complete comparison between the measures, despite the difference in scale. As it can be seen in Figure 6, both show an approximate similar behaviour, although the mtDNA in the NCD has a higher slope (taking into account the differences in scales, analogous to the NRC in Figure 5).

In primates, mtDNA is maternally inherited, while nuclear DNA in offspring represents a combination between mother and father. Although there are studies that show that the rates of nucleotide substitution vary among mitochondrial and nuclear DNAs [159], there is evidence that, in mammals, the spontaneous mutation rate in the germline is lower than in somatic cells [160]. The exact rates of evolution between the mtDNA and the whole gDNA in primates are not known, although it is widely believed that they are higher in mtDNA than in gDNA, given several sampling analysis [160]. Despite these studies have previously calculated mutation rates in both mtDNA and gDNA for primate species and showed that mtDNA mutation rates are higher than gDNA, these are mostly based in a subset of genes and include only coding regions. To our best knowledge, the analysis of whole genomes, including non-coding regions, has not been done so far and is presented in this paper using two measures.

Recombination and mobile genetic elements, such as retrotransposons, shape the genomic architecture, namely through large alteration events such as inversions, translocations, fusions, and fissions, being a major factor for genome reshuffling [161]. However, rearranged chromosomes present significantly lower recombination rates than chromosomes that have been maintained since the last common ancestor of great apes [162], mostly because inverted regions have lower recombination rates than collinear and noninverted regions, independently of the effect of centromeres [162]. Therefore, major alterations create an effect of evolutionary deceleration. For a compression-based analysis of inverted repeats in several species, see [163].

When comparing Figure 5 with Figure 6, we notice that the gDNA seems to have near half of the variation of the mtDNA, although with evidence of changing acceleration events, but always maintaining an equilibrium through time. When computing an evolutionary map (Figure 6B), we see that the NRC approximates the events of speciation in chimpanzee (a) with the gorilla (b) and orangutan (c) with the gibbon (d).

One of the applications of the relative compression is to localize and connect similar regions in DNA sequences through the computation of Equation (4). These regions are usually associated with rearrangements [71]. The conjoint information, combined with the information contained is not suitable for this purpose, namely because it does not ignore repetitive regions on the targets, originating a hard problem to delimitate the regions. On the other hand, the relative compression ignores repetitive regions in *x*, as Equation (8) shows.

**Figure 8:**
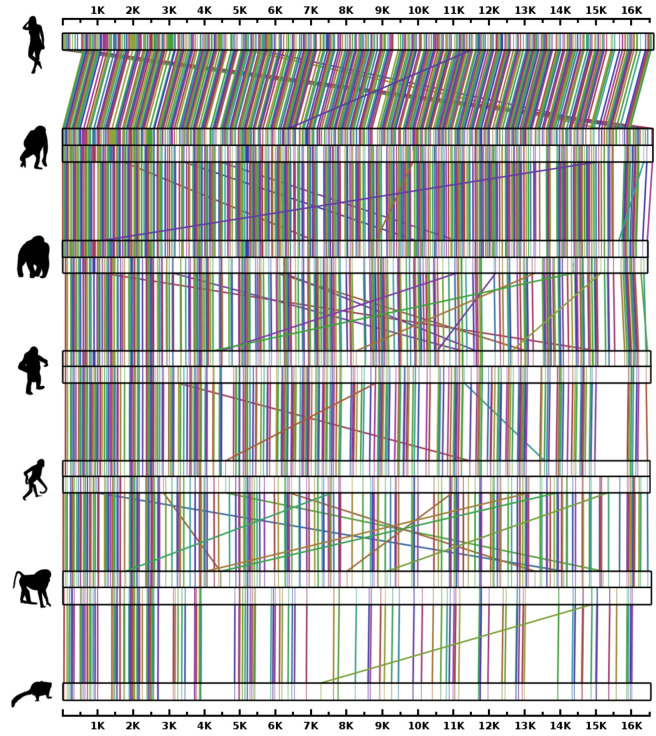
Patterns of similarity between mtDNA from different anthropoids, estimated with relative compression technology. From the top to the bottom: human-chimpanzee, chimpanzee-gorilla, gorilla-orangutan, orangutan-gibbon, gibbon-baboon, baboon-marmoset. The maps are depicted according to the output of the SMASH-contigs tool. This tool uses a simplified version of the computation of Equation (4). For replication use the script: runRearrange.sh.

In Figure 8, we use an approximation of the relative compression to compute relative similarities between the mtDNA of the seven primates, ordered by time of speciation. The figure shows a decrease in the number of connections while the time of speciation increases. Two reasons may be related to this. First, since the speciation time is higher, the variation accumulated is higher through time and, therefore, the similarity is lower. The second reason might be related to the size of the species, namely because small species seem to have higher evolution rates [164].

Interestingly, a region of the gorilla mtDNA has similarity with other regions, from distant region positions, relative to both chimpanzee and orangutan. The same happens in the gDNA, where one of the major rearrangement sources between the gorilla and both chimpanzee and orangutan is a chromosomal translocation between chromosomes 15 and five [153]. We recall that, besides the NUMTS, minimal correlations across both are known [165,166,167].

## 4. Discussion and Conclusions

In this paper, we have directly compared two measures, the Normalized Compression Distance (NCD) and the Normalized Relative Compression (NRC). Although related, both are two normalized measures to answer different questions. The NCD measures the information distance between two strings, independently from the direction. The NRC measures the information of a string *x* relatively to *y*, which may be different from measuring the information of *y* relatively to *x*.

As an analogy, consider that an observer is assisting to the run of one motorcyclist and a cyclist. The observer wants to quantify the amount of information needed to describe one given the other. The NCD aims to quantify the mutual information of both runners, accounting the amount of descriptive surprise that each runner has regarding the other, namely the use of a wheel, helmet, motor, pedal, etc. However, it only considers these elements once, since the amount of information between two elements, for example, wheels is minimal.

On the other hand, the NRC selects a runner as a reference, for example, the motorcyclist. After, models him (creating an informative representation of him), and, then, measures the amount of surprise that is seen on the cyclist, regardless if he has repetitive elements, such as two pedals. In this case, the information describing the repetitive features on the cyclist, that have not been seen in the motorcyclist, may be added to the final count after being multiplied by the first repetitive element seen. Therefore, the relative compression will ignore if the regions on the target are repetitive or not (Equation (8)). The normalization of the relative compression will, then, act as a rescaling operation, decreasing more the importance of the repetitions.

Notice that C(x∥y)≥C(x|y) and |x|≥C(x). The |x| usually represents a large quantity and, therefore, the NRC is usually less than the NCD, specially when the C(x) is much smaller than |x|. The absence of self-redundancy in the NRC and the relation between *x* and *y* seem to be the major factors for the NRC usually to be smaller than the NCD. In the real large examples (mRNA and gDNA), we have consistently found this.

Regarding computational time, the NRC uses, consistently, less time to be computed than the NCD. In the larger dataset (gDNA), we found that the computation of the NRC is roughly one-fifth of the NCD. Regarding memory, both can use the same memory, although the NCD needs more precision to store the memory model of the *x* and *y*, unlike the NRC that only needs to store the memory model of *y*.

The NRC is also able to efficiently detect and connect similar regions of information. The NRC is also able to produce a better adaptation, using lower computational resources, to the nature of the data given its simplicity. However, the NRC requires a specific relative compressor, while the NCD can be computed through the conjoint information, approximated by the concatenation of *x* and *y*.

We have compared both measures in genomic sequences with different scales and natures. These included mitochondrial DNA (mtDNA), messenger RNA (mRNA) and whole genome DNA (gDNA) of seven primates. With this approach, we provided several insights into evolutionary acceleration rates between different scales, namely a higher (near the double) variation rate of the mtDNA, relatively to the gDNA in these primates. Notice that the usage of alignment methods to estimate such measures in whole genomes is dependent on the capability to cope with genome rearrangements and high variation. In fact, quantifying dissimilarity of sequences with alignment methods is problematic, due to the need for fine-tuned thresholds, considering relaxed edit distances and, consequently, the increase of computational cost. More important, the choice of the thresholds have the problem of how to quantify dissimilarity without producing overestimated measures, namely measures that cope with an approximation of the information present and not an overestimation of the same value [73]. Therefore, we believe that compression-based normalized measures, independently of the use of NRC or NCD, are the natural measures to quantify such information.

Finally, we have shown a practical example of the relative compression for localizing similar information regions using the mtDNA of seven primates. This is an application where we have found that the relative mode question is more suitable for what we want to address.

## Figures and Tables

**Table 1:** Description of the dataset for the mtDNA, mRNA, and gDNA. All the sequences have been downloaded from the National Center for Biotechnology Information (NCBI).

Species	mtDNA		mRNA		gDNA	
	Length	Reference	Length	Version	Length	Version
Human	16,569	NC_012920.1	587,117,742	GRCh38.p7	2,948,627,755	GRCh38.p7
Chimpanzee	16,554	NC_001643.1	351,298,530	3.0	2,845,195,942	3.0
Gorilla	16,412	NC_011120.1	153,150,229	4	2,788,268,060	4
Orangutan	16,499	NC_002083.1	102,315,527	2.0.2	2,722,968,486	2.0.2
Gibbon	16,478	NC_021957.1	110,221,273	3.0	2,611,673,151	3.0
Baboon	16,516	NC_020006.2	312,140,410	3.0	2,727,993,489	3.0
Marmoset	16,499	NC_025586.1	172,133,747	3.2	2,618,690,967	3.2
Total	115,548	-	1,788,377,458	-	19,263,417,850	-

## Abbreviations

The following abbreviations are used in this manuscript:

NC	Normalized Compression
NCD	Normalized Compression Distance
NRC	Normalized Relative Compression
DNA	Deoxyribonucleic acid
mtDNA	mitochondrial DNA
gDNA	nuclear DNA
cpDNA	chloroplast DNA
mRNA	messenger Ribonucleic acid
SNP	Single Nucleotide Polymorphism
GeCo	Genomic Compressor (tool)
